# Socioeconomic and ethnic disparities associated with access to cochlear implantation for severe-to-profound hearing loss: A multicentre observational study of UK adults

**DOI:** 10.1371/journal.pmed.1004296

**Published:** 2024-04-04

**Authors:** Chloe Swords, Reshma Ghedia, Hannah Blanchford, James Arwyn–Jones, Elliot Heward, Kristijonas Milinis, John Hardman, Matthew E. Smith, Manohar Bance, Jameel Muzaffar, on behalf of INTEGRATE

**Affiliations:** 1 Cambridge Hearing Group, University of Cambridge, Cambridge, United Kingdom; 2 Cambridge University Hospitals NHS Foundation Trust, Cambridge, United Kingdom; 3 INTEGRATE (The Otolaryngology Trainee Research Network), United Kingdom; 4 Royal National ENT and Eastman Dental Hospitals, London, United Kingdom; 5 Great Ormond Street Hospital for Children NHS Foundation Trust, London, United Kingdom; 6 Charing Cross Hospital, London, United Kingdom; 7 Wythenshawe Hospital, Manchester, United Kingdom; 8 Liverpool University Hospitals NHS Foundation Trust, Liverpool, United Kingdom; 9 Royal Marsden Hospital, Fulham Road, London, United Kingdom

## Abstract

**Background:**

Patients with severe-to-profound hearing loss may benefit from management with cochlear implants. These patients need a referral to a cochlear implant team for further assessment and possible surgery. The referral pathway may result in varied access to hearing healthcare. This study aimed to explore referral patterns and whether there were any socioeconomic or ethnic associations with the likelihood of referral. The primary outcome was to determine factors influencing referral for implant assessment. The secondary outcome was to identify factors impacting whether healthcare professionals had discussed the option of referral.

**Methods and findings:**

A multicentre multidisciplinary observational study was conducted in secondary care Otolaryngology and Audiology units in Great Britain. Adults fulfilling NICE (2019) audiometric criteria for implant assessment were identified over a 6-month period between 1 July and 31 December 2021. Patient- and site-specific characteristics were extracted. Multivariable binary logistic regression was employed to compare a range of factors influencing the likelihood of implant discussion and referral including patient-specific (demographics, past medical history, and degree of hearing loss) and site-specific factors (cochlear implant champion and whether the hospital performed implants).

Hospitals across all 4 devolved nations of the UK were invited to participate, with data submitted from 36 urban hospitals across England, Scotland, and Wales. Nine hospitals (25%) conducted cochlear implant assessments. The majority of patients lived in England (*n* = 5,587, 86.2%); the rest lived in Wales (*n* = 419, 6.5%) and Scotland (*n* = 233, 3.6%). The mean patient age was 72 ± 19 years (mean ± standard deviation); 54% were male, and 75·3% of participants were white, 6·3% were Asian, 1·5% were black, 0·05% were mixed, and 4·6% were self-defined as a different ethnicity.

Of 6,482 submitted patients meeting pure tone audiometric thresholds for cochlear implantation, 311 already had a cochlear implant. Of the remaining 6,171, 35.7% were informed they were eligible for an implant, but only 9.7% were referred for assessment. When adjusted for site- and patient-specific factors, stand-out findings included that adults were less likely to be referred if they lived in more deprived area decile within Indices of Multiple Deprivation (4th (odds ratio (OR): 2·19; 95% confidence interval (CI): [1·31, 3·66]; *p* = 0·002), 5th (2·02; [1·21, 3·38]; *p* = 0·05), 6th (2·32; [1·41, 3·83]; *p* = 0.05), and 8th (2·07; [1·25, 3·42]; *p* = 0·004)), lived in London (0·40; [0·29, 0·57]; *p* < 0·001), were male (females 1·52; [1·27, 1·81]; *p* < 0·001), or were older (0·97; [0·96, 0·97]; *p* < 0·001). They were less likely to be informed of their potential eligibility if they lived in more deprived areas (4th (1·99; [1·49, 2·66]; *p* < 0·001), 5th (1·75; [1·31, 2·33], *p* < 0·001), 6th (1·85; [1·39, 2·45]; *p* < 0·001), 7th (1·66; [1·25, 2·21]; *p* < 0·001), and 8th (1·74; [1·31, 2·31]; *p* < 0·001) deciles), the North of England or London (North 0·74; [0·62, 0·89]; *p* = 0·001; London 0·44; [0·35, 0·56]; *p* < 0·001), were of Asian or black ethnic backgrounds compared to white patients (Asian 0·58; [0·43, 0·79]; *p* < 0·001; black 0·56; [0·34, 0·92]; *p* = 0·021), were male (females 1·46; [1·31, 1·62]; *p* < 0·001), or were older (0·98; [0·98, 0·98]; *p* < 0·001).

The study methodology was limited by its observational nature, reliance on accurate documentation of the referring service, and potential underrepresentation of certain demographic groups.

**Conclusions:**

The majority of adults meeting pure tone audiometric threshold criteria for cochlear implantation are currently not appropriately referred for assessment. There is scope to target underrepresented patient groups to improve referral rates. Future research should engage stakeholders to explore the reasons behind the disparities. Implementing straightforward measures, such as educational initiatives and automated pop-up tools for immediate identification, can help streamline the referral process.

## Abstract 2

In this multi-centre UK wide study including >6000 participants, Chloe Swords and co-workers explore referral patterns for cochlear implantation vary across the UK according to ethnicity and socioeconomics.

## Author summary

**Why was this study done?:**

• This study was undertaken to investigate referral patterns for cochlear implantation among patients meeting pure tone audiometric threshold criteria in Great Britain and to identify if socioeconomic or ethnic disparities effect the likelihood of referral.

**What did the researchers do and find?:**

• Researchers conducted an observational study across 36 secondary care Otolaryngology and Audiology units in Great Britain, focusing on adults who met pure tone audiometric threshold criteria for cochlear implant assessment.

• Of 6,171 included adults identified, 36% were informed of their potential eligibility for a cochlear implant, but only 9% were actually referred for assessment.

• Referral likelihood was lower for those in more deprived areas, living in London, of male sex, or older age.

• Ethnic minorities, particularly of Asian or black backgrounds, were also less likely to be informed about their eligibility.

**What do these findings mean?:**

• These data suggest that the majority of adults meeting pure tone audiometric threshold criteria for cochlear implantation are not being referred appropriately for cochlear implant assessments, indicating significant disparities in patient management based on socioeconomic status, ethnicity, and sex.

• These disparities suggest a need for targeted efforts to improve referral rates among underrepresented groups and to ensure all patients are informed of their treatment options.

• Further research is required to understand and address these disparities, with initiatives to ensure informed decision-making, educate healthcare providers, and explore reasons for declining cochlear implant assessments, especially among older ethnic minority groups.

• Study limitations include its observational retrospective nature, potential inaccuracies in the reporting of sex and gender, and the impact of the COVID-19 pandemic on referral protocols.

## Introduction

In the United Kingdom (UK), one in 5 adults experiences hearing loss, with approximately 1·2 million people suffering from severe–to–profound loss [1]. By 2030, hearing loss is expected to be a top 10 health burden, costing the UK £38·6 billion per year [2]. A cochlear implant (CI), which bypasses inner ear damage providing a clearer auditory signal to the brain (compared to hearing aids), is an effective treatment for this degree of hearing loss [3]. Implantation has consistently been demonstrated to improve patient quality of life [4].

Patients receiving treatment from the National Health Service (NHS) in the UK would typically require their primary care practitioner to make a referral to Otolaryngology or Audiology secondary care service for their hearing or other otological symptoms. In the secondary care setting, a pure tone audiogram can be arranged. If the patient meets the pure tone audiometric threshold criteria for cochlear implantation, they should have a discussion about their potential eligibility for cochlear implantation. If the patient agrees, they can be referred to a cochlear implant centre where a multidisciplinary team will conduct further detailed audiological testing including speech audiometry and clinical evaluation before proceeding with surgery and rehabilitation. Speech audiometry is not routinely performed for standard audiological assessment for hearing loss. There are only 20 cochlear implant centres across the UK. Many patients are routinely under audiology services to fit and maintain their conventional hearing aids and can be referred for cochlear implantation if their hearing deteriorates.

In the UK, the National Institute for Health and Care Excellence (NICE) has recommended that unilateral cochlear implantation is considered for adults who have severe-to-profound hearing loss. In March 2019, the definition of severe-to-profound hearing loss was updated by NICE to be greater than or equal to 80 dBHL at 2 or more frequencies. Prior to this update, the definition was greater than or equal to 90 dBHL at 2 or more frequencies.

Despite the reported benefits of implantation, it has been recognised that many adults who could benefit are never implanted [5]. The implantation rate is thought to be as low as 5% to 10% of those potentially eligible [6], although there has been no recent assessment of this across the UK. Although disparities have been documented in hearing healthcare and implant uptake, the majority of studies evaluating access to implants focus on what happens once patients are assessed at implant centres, rather than the stages leading up to and including referral for assessment [7,8]. Analysing patterns at these gateway stages in a patient’s cochlear implant journey, may lead to a deeper understanding of the referral pathway, enabling identification of the main drivers of inequalities in care, as well as the impact of key healthcare disparities, such as ethnicity and socioeconomic status.

The first aim of the study was to examine what proportion of patients who had met the pure tone audiometric threshold criteria for cochlear implantation as per the NICE guidelines had discussion about their eligibility and referred for further assessment by a cochlear implant team. The second aim was to identify if any determinants of socioeconomic and ethnic inequalities were associated with referral for these patients. This was done via a multicentre national assessment of eligible patients meeting the pure tone audiometric threshold criteria. Our working hypothesis posited that not all eligible patients are consistently referred for assessment and that referral rates may differ across ethnic or socioeconomic cohorts. Assessing disparities between patients who had a symptom of hearing loss and sought medical care to those who did not, and those who were referred from primary care to secondary care and those who were not was outside the scope of this study.

## Methods

This study is reported as per the Strengthening the Reporting of Observational Studies in Epidemiology (STROBE) guideline (S1 Checklist). The study protocol was published in advance (https://entintegrate.co.uk).

### Ethical considerations

The Health Research Authority decision tool determined that the study design fell under the remit of a service evaluation, and so no ethical approval was required (available at http://hra-decisiontools.org.uk/research/).

### Study design and population

A study was conducted to identify all adults with severe-to-profound hearing loss who were eligible for a cochlear implant based on pure tone threshold criteria. This meant only patients seen in secondary care audiology were able to be included due to the methodology. The inclusion criteria were adults (18 years and older) who had audiometric testing (pure-tone audiometry, auditory brainstem response or comparable) between 1 July and 31 December 2021 that confirmed their eligibility for referral after completing audiometric evaluation as per the revised NICE criteria [3]. Eligible audiometric thresholds were defined as hearing only sounds that were louder than 80 decibel hearing level (dBHL) at 2 or more frequencies (0.5/1/2/3/4 kHz) bilaterally when unaided [3]. To promote homogeneity, data were only accepted from centres with Auditbase software (Auditdata, Copenhagen, Denmark), the most commonly used UK audiology software.

The study was delivered by INTEGRATE (UK Otolaryngology Trainee Research Network), and supported by the British Society of Otology (BSO), and British Cochlear Implant Group (BCIG). All adult UK general otolaryngology, otology, audiology, and audiovestibular departments were invited to participate via social media and mailouts from supporting organisations (potential of 174 sites). Each participating site designated a site lead and registered the study in accordance with local clinical governance guidelines. The Health Research Authority decision tool determined that the study fell under the remit of a service evaluation, and so no ethical approval was required (https://hra–decisiontools.org.uk/research/). Individual management and follow-up was performed by the site team; the project management team only handled anonymised data.

### Case identification and collection

Eligible cases were identified on Auditbase, an audiology database, using an open-source search tool, as designed by the BCIG (https://www.bcig.org.uk/champions_scheme.aspx). Clinical and audiometric data were collected by local teams using a standardised form. Data was collected from the Auditbase audiological electronic health record. If data was missing, then further information was gathered from the clinical hospital electronic health record. Anonymised forms were submitted to the project management team for analysis and de-duplication (S1 Appendix).

Sites converted patients’ postcode to Indices of Multiple Deprivation (IMD) ranks and deciles prior to submission. Missing or ambiguous data were clarified with local sites to ensure data completeness. Individual cases were not included if primary outcome data were missing. Missing individual predictor variable data was not a criterion for exclusion.

### Primary and secondary study outcomes

The primary outcome was to determine the factors influencing referral after completing audiometric evaluation for implant assessment. The secondary outcome was to identify the factors impacting on whether healthcare professionals had discussed the option of referral after completing audiometric evaluation with the patient.

Primary and secondary outcomes and predictor variables were identified through an examination of patient case notes, communications with primary care providers, and referral letters sent to tertiary cochlear implant providers. This information was identified by either Auditbase or the hospital clinical health records.

### Predictor variables

The predictors of interest were socioeconomic and ethnicity measures. Socioeconomic measures were assessed according to the patient’s home postcode, which provided information regarding the IMD decile and geographic region that adults lived in within the UK, according to NHS (England). Further information regarding interpretation of IMD is provided in S2 Appendix. In NHS healthcare records, ethnicity is coded according to the 2021 UK census categories. This includes 5 categories (white, Asian, black, mixed, other), which are further subcategorised into 19 subgroups. Patients select their chosen ethnicity, which is then stored on their healthcare record. We also recorded whether English was the patient’s first language; again this is a patient-reported measure.

Other patient characteristics (demographic and healthcare) were extracted from patient healthcare records and were chosen on the clinical basis as well as published literature. Variables included presence of multimorbidity (2 or more long-term health conditions), existence of learning disabilities, physical disabilities, cognitive impairment, visual impairment, history of meningitis, and degree of hearing loss (the severity boundary was defined as greater than 90 dBHL hearing threshold in line with NICE 2009 referral guidelines). Hospital factors (presence of cochlear implant champion at the hospital and whether the hospital was an implant centre) were collected by administering a questionnaire to site leads. Cochlear implant champions are local clinicians (often audiologists) who have volunteered for this position and have been trained through the British Academy of Audiology and BCIG to promote implant awareness (https://www.baaudiology.org/professional-information/cochlear-implant-champions/). Their role is to train, support, and empower local team members to counsel patients and their families about cochlear implants and ensure that all eligible patients who meet pure tone audiometric threshold criteria are given the information needed to make an informed decision about referral after completing audiometric evaluation. Additionally, cochlear implant champions are responsible for monitoring and auditing cochlear implant pathways in their service and dedicate 1 h of protected time per week to this process.

These variables were selected following a literature review to identify potentially important confounding variables. The variables were discussed with a Scientific Expert Advisory Group, comprising 18 experts in the field of Otolaryngology, Audiology, and Hearing science. This enabled the incorporation of confounding variables into the multivariable regression model.

### Statistics

Descriptive statistics were used to describe the proportions of eligible patients referred or discussed, and the characteristics of included patients. Categorical variables were analysed as non-ordered nominal variables. Baseline characteristics were described as means or proportions. They were compared across categories of ethnic groups using chi-squared tests for categorical variables and one-way analysis of variance for continuous variables.

Logistic regression modelling was planned to assess the relationship of variables with the primary and secondary outcomes, without (simple logistic) and with (multiple backwards stepwise) adjustments for socioeconomic, ethnicity, clinical, and hospital factors. Where possible, for categorical variables the reference value was defined by not having the condition; for other categorical variables, categories with the greatest proportion of adults submitted were selected (white, male adults, and the Midlands and East Region). In the case of IMD, we were most interested in whether patients who live in the most deprived locations were less likely to be referred; hence, for this category IMD score one was designated as the reference. As direct comparisons between the 4 constituent nations of the UK were not possible for IMD [9], only adults residing in England were considered for the multivariable adjusted regression model. Data were presented as odds ratios (OR) and 95% confidence intervals (95% CI).

Statistical analyses were conducted using SPSS software version 28.0 (IBM, Chicago, United States of America). A two-sided *p*-value of <0·05 was considered statistically significant. Sample size calculations were not performed; all eligible submitted data within the timeframe were accepted.

### Exploratory and sensitivity analysis

Exploratory analyses using the adjusted multivariable model were conducted for the relevant deprivation domains that comprised the IMD decile rank: health, education, barriers to housing and services, as well as the income deprivation affecting older people index (IDAOPI). These data were only available for adults with an English postcode (*n* = 5,587).

Some data were missing and were coded as “unknown.” Sensitivity analyses were performed where adults with missing data were excluded to examine the potential for information and confounding bias. Further analysis of missing data was performed by replacing missing data using multiple imputation in SPSS.

### Patient and public involvement

Patients often express to us that they wish they had been told about the option of implantation earlier and have expressed concern that other patients may “miss out” due to geographical, socioeconomic, ethnic, or other factors. While not directly involved in the methodology of this study, patients and their families are actively involved in our group’s follow-on work to address the inequalities highlighted in this study.

## Results

### Baseline patient characteristics

**Fig 1:**
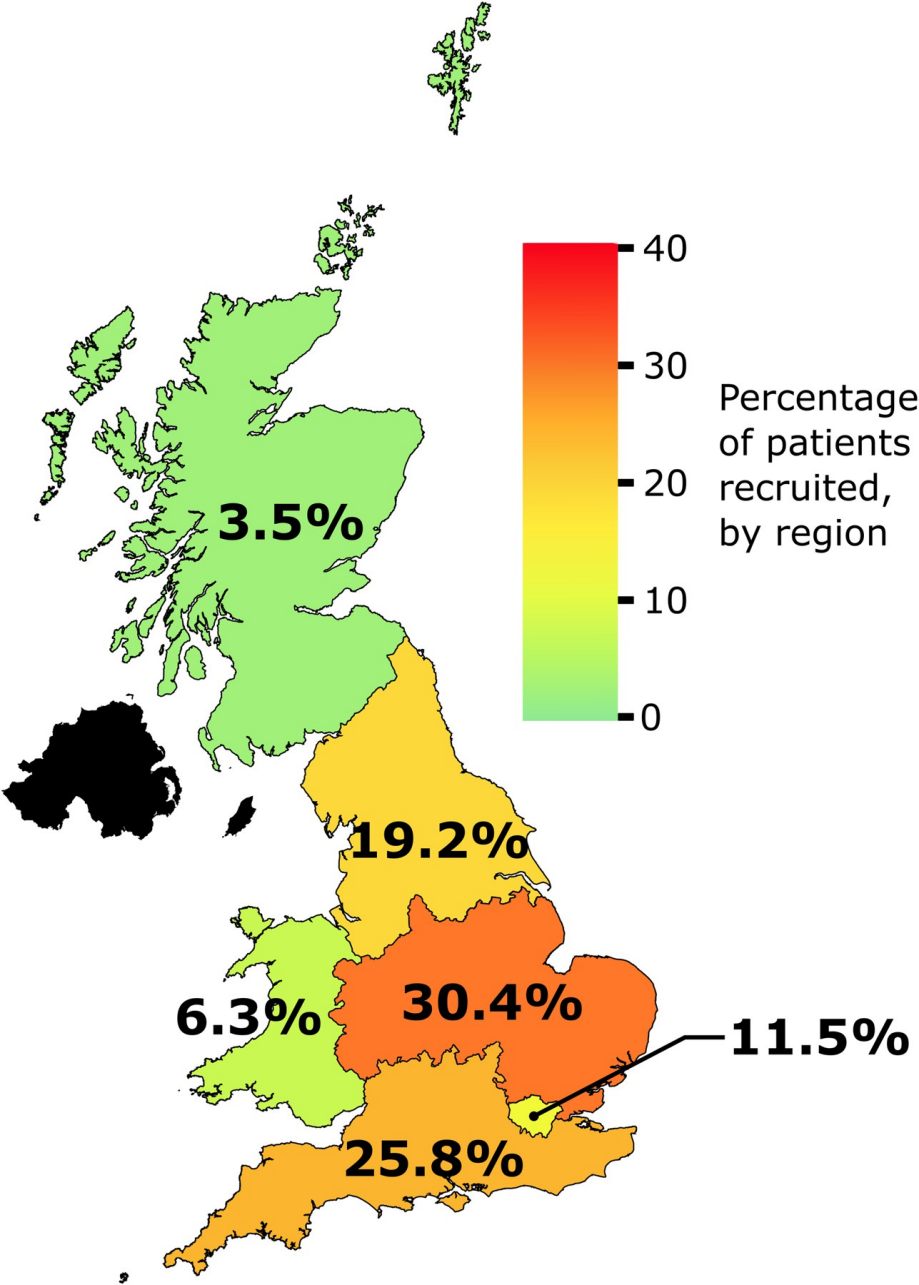
Map of the number of adults submitted to the study according to the region of residence. Black shading indicates data was not submitted from this region. The image of the UK was adapted from mapSVG under the creative commons license (https://mapsvg.com/maps/united-kingdom).

Data from 36 hospitals across England, Wales, and Scotland were submitted, representing 6,760 adults (**Fig 1**). No hospitals in Northern Ireland participated. The largest proportion of data submitted was from centres in the Midlands and East of England region (30·4%). In total, 80 hospitals registered their interest; 24 were not eligible for involvement due to noncompatible software and 20 withdrew following registration.

**Fig 2:**
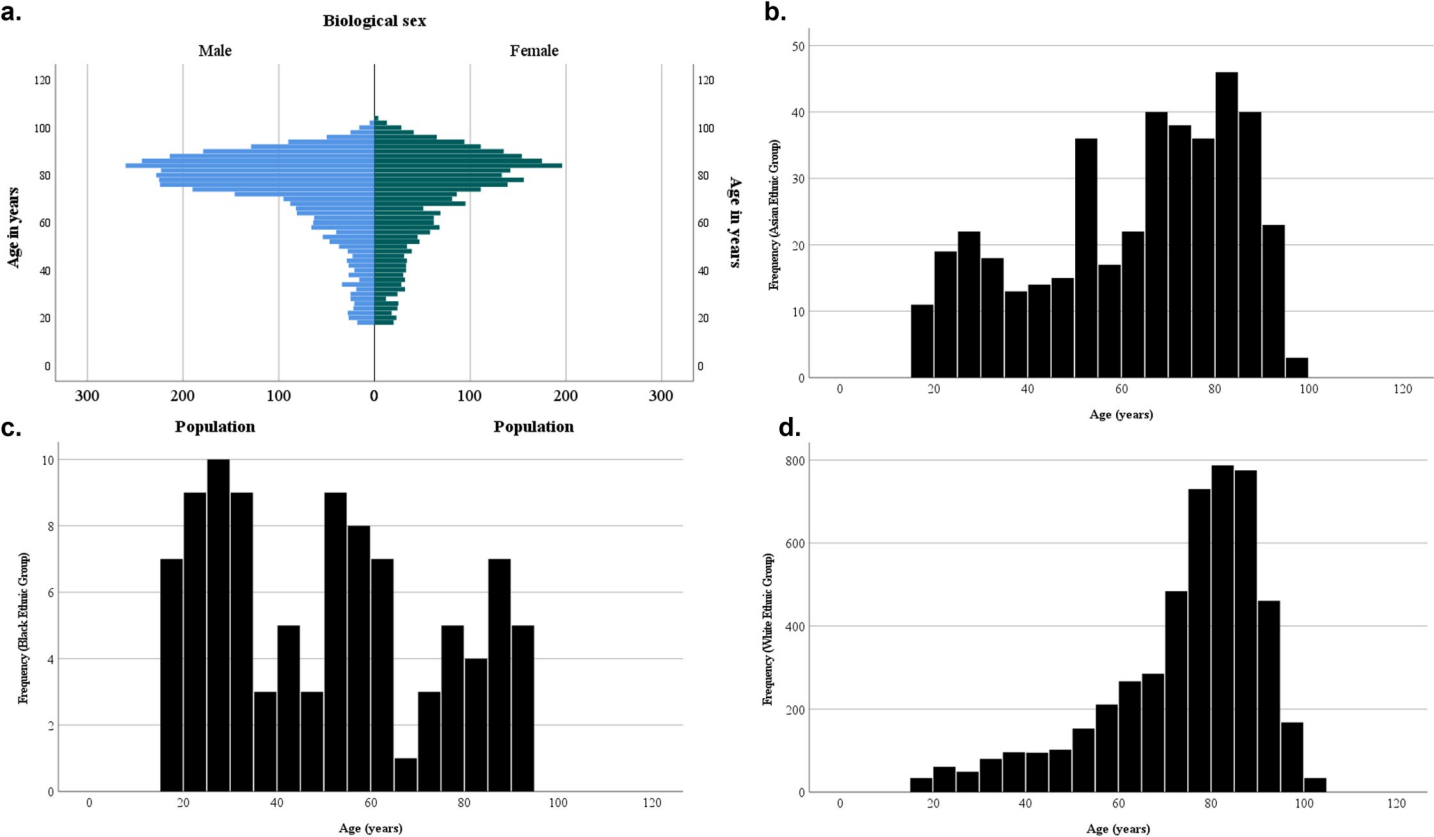
(a) Population pyramid by age and sex assigned at birth. (b–d) Population age distribution of Asian (b), black (c), and white (d) adults.

Following implementation of inclusion criteria and de-duplication of adults, 6,482 adults met pure tone audiological threshold criteria (S1 Flowchart). The baseline characteristics of the included adults are shown in **Table 1**. The mean age was 72 ± 19 years (mean ± standard deviation); 53 9% of adults were male (**Fig 2A**), and 75·3% of participants were white, 6·3% were Asian, 1·5% were black, 0·05% were mixed, and 4·6% were self-defined as a different ethnicity. Ethnicity data were missing in 11·8% of participants. As compared to the other ethnic groups (Asian, black, mixed, other), white adults were older (62, 50, 58, 64 versus white 74 years, respectively) (**Fig 2B**). The age distributions of white and Asian adults were skewed to the left. Age (continuous variable) was moderately positively associated with presence of multimorbidity (binary variable) (Pearson point-biserial correlation coefficient r = 0.3; *p* < 0.001), and 311 adults who already had a cochlear implant were submitted, leaving 6,171 eligible adults for inclusion in the regression analysis (S1 Flowchart).

**Table 1:** Baseline characteristics of included patients, stratified by ethnicity. Data are presented as *n* (column %) or mean [95% CI], other than age (mean ± SD). dBHL: decibel hearing level. Values are given prior to removal of implanted patients.

Characteristic	Asian (*n* = 408)	Black (*n* = 95)	Mixed (*n* = 33)	Other (*n* = 300)	Missing (*n* = 767)	White (*n* = 4,879)	Total (*n* = 6,482)
Age, mean [SD], years	61·91 [21·83]	50·28 [23·55]	58·44 [23·35]	64·18 [20·94]	72·56 [18·67]	73·65 [17·30]	71·93 [18·63]
Sex							
Male	220 (53·9)	41 (43·2)	14 (42·4)	154 (51·3)	441 (57·5)	2,641 (40·7)	3,511 (54·2)
Female	181 (44·4)	53 (55·8)	19 (57·6)	144 (48)	316 (41·2)	2,217 (34·2)	2,930 (45·2)
Missing	7 (1·7)	1 (1·1)	0 (0)	2 (0·7)	10 (1·3)	21 (0·3)	41 (0·6)
Index of multiple deprivation decile							
1 (most deprived)	74 (18·1)	11 (11·6)	4 (12·1)	41 (13·7)	63 (8·2)	426 (8·7)	619 (9·5)
2	79 (19·4)	24 (25·3)	9 (27·3)	34 (11·3)	49 (6·4)	432 (8·9)	627 (9·7)
3	38 (9·3)	18 (18·9)	2 (6·1)	22 (7·3)	46 (6)	411 (8·4)	537 (8·3)
4	47 (11·5)	14 (14·7)	4 (12·1)	32 (10·7)	73 (9·5)	412 (8·4)	582 (9)
5	46 (11·3)	5 (5·3)	4 (12·1)	32 (10·7)	83 (10·8)	484 (9·9)	654 (10·1)
6	43 (10·5)	9 (9·5)	2 (6·1)	28 (9·3)	105 (13·7)	458 (9·4)	645 (10)
7	17 (4·2)	4 (4·2)	2 (6·1)	28 (9·3)	76 (9·9)	513 (10·5)	640 (9·9)
8	23 (5·6)	4 (4·2)	1 (3)	30 (10)	93 (12·1)	530 (10·9)	681 (10·5)
9	18 (4·4)	1 (1·1)	1 (3)	21 (7)	78 (10·2)	562 (11·5)	681 (10·5)
10 (least deprived)	16 (3·9)	3 (3·2)	3 (9·1)	29 (9·7)	68 (8·9)	606 (12·4)	725 (11·2)
Missing	7 (1·7)	2 (2·1)	1 (3·0)	3 (1·0)	33 (4·3)	45 (0·9)	91 (1·4)
Region							
Midlands and East	177 (43·4)	20 (21·1)	12 (27·3)	130 (43·3)	431 (56·2)	1,224 (25·1)	1,994 (30·8)
London	136 (33·3)	51 (53·7)	7 (12·1)	84 (28)	20 (2·6)	454 (9·3)	752 (11·6)
North	32 (7·8)	4 (4·2)	1 (12·1)	16 (5·3)	27 (3·5)	1,116 (22·9)	1,196 (18·5)
South	33 (8·1)	14 (14·7)	12 (6·1)	56 (18·7)	31 (4·0)	1,499 (30·7)	1,645 (25·4)
Scotland	5 (1·2)	0 (0·0)	1 (12·1)	1 (0·3)	0 (0·0)	226 (4·6)	233 (3·6)
Wales	7 (1·7)	1 (1·1)	0 (6·1)	10 (3·3)	221 (28·8)	180 (3·7)	419 (6·5)
Missing	18 (4·4)	5 (5·3)	0 (6·1)	3 (1·0)	37 (4·8)	180 (3·7)	243 (3·7)
Was English the patients first language?							
Yes	98 (24)	57 (60)	20 (6·0)	80 (26·7)	420 (54·8)	4,585 (70·7)	5,260 (81·1)
No	192 (47·1)	19 (20)	6 (1·8)	58 (19·3)	16 (2·1)	124 (1·9)	415 (6·4)
Missing	118 (28·9)	19 (20)	7 (2·1)	162 (54)	331 (43·2)	170 (2·6)	807 (12·4)
Was the hearing test performed at a cochlear implant centre?							
Yes	149 (36·5)	65 (68·4)	15 (45·5)	137 (45·7)	155 (20·2)	1,621 (33·2)	2,142 (33)
No	259 (63·5)	30 (31·6)	18 (54·6)	163 (54·3)	612 (79·8)	3,259 (66·8)	4,340 (66·9)
Multimorbidity							
Yes	116 (28·4)	24 (25·3)	8 (24·2)	89 (29·7)	206 (26·9)	2,017 (41·3)	2,460 (38)
No	235 (57·6)	57 (60)	20 (60·6)	176 (58·7)	249 (32·5)	2,236 (45·8)	2,973 (45·9)
Missing	57 (14·0)	14 (14·7)	5 (15·2)	35 (11·7)	312 (40·7)	627 (12·8)	1,049 (16·2)
Learning disability							
Yes	13 (3·2)	4 (4·2)	0 (0·0)	8 (2·7)	11 (1·4)	118 (2·4)	154 (2·4)
No	369 (90·4)	83 (87·4)	33 (100·0)	264 (88·0)	447 (58·3)	4,459 (91·4)	5,655 (87·2)
Missing	26 (6·4)	8 (8·4)	0 (0·0)	28 (9·3)	309 (40·3)	302 (6·2)	673 (10·4)
Cognitive disability							
Yes	16 (3·9)	3 (3·2)	1 (3·0)	14 (4·7)	42 (5·5)	356 (7·3)	432 (6·7)
No	371 (90·9)	86 (90·5)	32 (97·0)	257 (85·7)	401 (52·3)	4,024 (82·5)	5,171 (79·8)
Missing	21 (5·1)	6 (6·3)	0 (0·0)	29 (9·7)	324 (42·2)	500 (10·2)	880 (13·6)
Visual impairment							
Yes	36 (8·8)	11 (11·6)	5 (15·2)	17 (5·7)	98 (12·8)	726 (14·9)	893 (13·8)
No	343 (84·1)	75 (78·9)	27 (81·8)	244 (81·3)	365 (47·6)	3,620 (74·2)	4,674 (72·1)
Missing	29 (7·1)	9 (9·5)	1 (3·0)	39 (13·0)	304 (39·6)	533 (10·9)	915 (14·1)
Physical disability							
Yes	39 (9·6)	6 (6·3)	1 (3·0)	27 (9·0)	36 (4·7)	662 (13·6)	771 (11·9)
No	335 (82·1)	81 (85·3)	27 (81·8)	245 (81·7)	408 (53·2)	3,513 (72·0)	4,609 (71·1)
Missing	34 (8·3)	8 (8·4)	4 (12·1)	28 (9·3)	323 (42·1)	704 (14·4)	1,101 (17)
Hearing thresholds worse than 90 decibels hearing level							
Yes	380 (93·1)	91 (95·8)	31 (93·9)	283 (94·3)	712 (92·8)	4,573 (93·7)	6,070 (93·6)
No	28 (6·9)	4 (4·2)	2 (6·1)	17 (5·7)	55 (7·2)	306 (6·3)	412 (6·4)
Meningitis							
Yes	2 (0·5)	4 (4·2)	0 (0·0)	2 (0·7)	1 (0·1)	28 (0·6)	37 (0·6)
No	345 (84·6)	75 (78·9)	26 (78·8)	256 (85·3)	440 (57·4)	3,902 (80·0)	5,044 (77·8)
Missing	61 (15·0)	16 (16·8)	6 (18·2)	42 (14)	326 (42·5)	949 (19·5)	1,400 (21·6)
Was a cochlear implant champion present at the hospital?							
Yes	365 (89·5)	83 (87·4)	25 (75·8)	270 (90·0)	546 (71·2)	3,734 (76·5)	5,023 (77·5)
No	43 (10·5)	12 (12·6)	7 (21·2)	30 (10·0)	221 (28·8)	1,145 (23·5)	1,458 (22·5)

### Baseline hospital characteristics

Out of 36 hospitals submitting data, 9 (25%) conducted tertiary cochlear implant assessments and surgery for adults with severe-to-profound hearing loss. A cochlear implant champion was present in 27 (75%) of submitting hospitals, but the amount of dedicated nonclinical time varied between hospitals from zero hours/unspecified per month (15 sites, representing 2,723 adults) to 2 h or fewer (4 sites/921 adults), to greater than 2 h (8 sites/1,593 adults).

### Primary outcome

#### Association of ethnicity and socioeconomic predictors on likelihood of referral for CI

Overall, 9.7% (*n* = 546/6,171) of eligible adults meeting pure tone audiological threshold criteria were referred for implantation assessment. The results of the logistic regression models for the association of predictor variables are shown in **Table 2**. In the fully adjusted model, the deprivation index of adults was significantly associated with likelihood of referral after completing audiometric evaluation, with adults from the least deprived locations being more likely to be referred than those from the most deprived. This association was strongest for adults from the 4th (OR: 2·19; 95% CI: [1·31, 3·66]; *p* = 0.002), 5th (OR: 2·32; [1·41, 3·83]; *p* = 0.005), 6th (OR: 2·32; [1·41, 3·83]; *p* < 0.001), and 8th deciles (OR: 2·07; [1·25, 3·42]; *p* = 0.004), where 10th is the least deprived region. There were significant regional differences, with adults living in London (OR: 0·40; [0·29, 0·57]; *p* < 0.001) being less likely to be referred after completing audiometric evaluation compared to adults from the Midlands and East. Ethnicity was not significantly associated with likelihood for referral.

**Table 2:** Results of simple and multivariable logistic regression analysis for the primary outcome: whether patients were referred for surgical management of their hearing or not.

Characteristic	No. of events, *n/N* (%)	Simple logistic regression			Multivariable logistic adjusted model*		
		Beta	OR [95% CI]	*p-value*	Beta	OR [95% CI]	*p-value*
**Ethnicity and socioeconomic factors**							
**Race/ethnicity**				<0·001			<0·001
White	375/4,679 (8.0)	Reference			Reference		
Asian	30/385 (7.8)	−0·031	0·97 [0·66, 1·43]	0·877	−0·34	0·71 [0·44, 1·14]	0·160
Black	13/89 (14.6)	0·68	1·96 [1·08, 3·57]	0·027	−0·39	0·69 [0·35, 1·34]	0·263
Mixed	3/28 (10.7)	0·32	1·38 [0·41, 4·58]	0·602	−0·49	0·62 [0·17, 2·30]	0·470
Other	31/280 (11.1)	0·36	1·43 [0·97, 2·11]	0·071	−0·31	0·73 [0·46, 1·18]	0·200
Missing	94/710 (13.2)	0·56	1·75 [1·38, 2·23]	<0·001	0·90	2·43 [1·74, 3·41]	<0·001
**IMD decile**				<0·001			<0·001
1st (most deprived)	34/608 (5.6)	Reference			Reference		
2nd	49/601 (8.2)	0·41	1·50 [0·95, 2·36]	0·061	0·46	1·59 [0·94, 2·69]	0·070
3rd	47/504 (9.3)	0·55	1·74 [1·10, 2·75]	0·015	0·49	1·64 [0·95, 2·83]	0·063
4th	64/553 (11.6)	0·80	2·21 [1·43, 3·41]	<0·001	0·78	2·19 [1·31, 3·66]	0·002
5th	59/626 (9.4)	0·56	1·76 [1·13, 2·72]	0·009	0·70	2·02 [1·21, 3·38]	0·005
6th	75/616 (12.2)	0·85	2·34 [1·54, 3·57]	<0·001	0·84	2·32 [1·41, 3·83]	<0·001
7th	55/618 (8.9)	0·50	1·65 [1·06, 2·57]	0·031	0·38	1·46 [0·86, 2·46]	0·154
8th	65/652 (10.0)	0·63	1·87 [1·22, 2·88]	0·004	0·73	2·07 [1·25, 3·42]	0·004
9th	42/645 (6.5)	0·16	1·18 [0·74, 1·88]	0·517	0·16	1·17 [0·68, 2·03]	0·548
10th (least deprived)	47/689 (6.8)	0·21	1·24 [0·78, 1·95]	0·232	0·23	1·26 [0·74, 2·15]	0·274
**Region**				<0·001			
Midlands and East	196/1,923 (10.2)	Reference			Reference		
London	69/654 (10.6)	0·04	1·04 [0·78, 1·39]	0·795	−0.93	0.40 [0·28, 0·57]	<0.001
North	51/1,189 (4.3)	−0·93	0·40 [0·29, 0·54]	<0·001	−0.34	0.71 [0·50, 1.02]	0.061
South	174/1,565 (11.1)	0·10	1·10 [0·89, 1·37]	0·377	−0.004	1.00 [0·76, 1.31]	0.977
Scotland	9/228 (3.9)	−1·02	0·36 [0·18, 0·72]	0·004	n/a		
Wales	34/400 (8.5)	−0·20	0·82 [0·56, 1·20]	0·303	n/a		
Missing	13/212 (6.1)	−0·55	0·58 [0·32, 1·03]	0·062	0.07	1.07 [0·52, 2.22]	0.854
**Healthcare factors**							
**Multimorbidity**				<0·001			
No	313/2,784 (11.2)	Reference					
Yes	158/2,404 (6.6)	−0·59	0·55 [0·46, 0·68]	<0·001			
Missing	75/983 (7.6)	−0·43	0·65 [0·50, 0·85]	0·001			
**Cognitive disability**				<0·001			
No	486/4,925	Reference					
Yes	16/424 (3.8)	−1·03	0·36 [0·22, 0·60]	<0·001			
Missing	44/822 (5.4)	−0·66	0·52 [0·38, 0·71]	<0·001			
**Physical disability**				<0·001			
No	427/4,376 (9.8)	Reference					
Yes	47/759 (6.2)	−0·49	0·61 [0·45, 0·83]	0·002			
Missing	72/1,036 (6.9)	−0·37	0·69 [0·53, 0·90]	0·005			
**Meningitis**				<0·001			
No	466/4,816 (9.7)	Reference					
Yes	3/34 (8.8)	−0·10	0·90 [0·28, 2·97]	0·848			
Missing	77/1,321 (5.8)	−0·55	0·58 [0·45, 0·74]	<0·001			
**Visual impairment**				0·045			
No	415/4,474 (9.3)	Reference					
Yes	77/861 (8.9)	−0·04	0·96 [0·74, 1·24]	0·757			
Missing	54/836 (6.5)	−0·39	0·68 [0·50, 0·91]	0·009			
**Learning disability**							
No	486/5,403 (9.0)	Reference					
Yes	16/150 (10.7)	0·19	1·21 [0·71, 2·05]	0·482			
Missing	44/618 (7.1)	−0·25	0·78 [0·56, 1·07]	0·120			
**Sex**				<0·001			
Male	245/3,355 (7.3)	Reference					
Female	298/2,787 (10.7)	0·42	1·52 [1·27, 1·81]	<0·001			
Missing	3/29 (10.3)	0·38	1·47 [0·44, 4·87]	0·534			
**Age**							
	Referred 61 year; Not referred 74 year	−0·03	0·97 [0·96, 0·97]	<0·001			
**Hearing thresholds worse than 90 decibels hearing level**							
No	10/407 (2.5)	Reference					
Yes	536/5,764 (9.3)	1·40	4·07 [2·16, 7·67]	<0·001			
**English as 1st language**				0·004			
No	44/382 (11.5)	Reference					
Yes	418/5,048 (8.3)	−0·37	0·69 [0·50, 0·96]	0·030			
Missing	84/741 (11.3)	−0·02	0·98 [0·67, 1·45]	0·927			
**Hospital factors**							
**Cochlear implant centre co-located**							
No	181/4,294 (4.2)	Reference					
Yes	365/1,877 (19.4)	1·70	5·49 [4·55, 6·62]	<0·001			
**Cochlear implant champion**							
No	146/1,431 (10.2)	Reference					
Yes	400/4,740 (8.4)	−0·21	0·81 [0·66, 0·99]	0·040			

* These analyses were run for patients with an English postcode only as IMD deciles are not comparable across the devolved nations; the majority (86%) of patients had an English postcode. Total included patients, *N* = 6,171.

CI, confidence interval; IMD, indices of multiple deprivation; OR, odds ratio.

### Association of other predictors on likelihood of referral after completing audiometric evaluation for cochlear implant

In the unadjusted model, the age of adults was significantly associated with likelihood of referral after completing audiometric evaluation (*p* < 0·001), with older adults being less likely to be referred (OR: 0·97; [0·96, 0·97]; *p* < 0.001). Female adults were more likely to be referred (OR: 1·52; [1·27, 1·81]; *p* < 0.001). Adults with multimorbidity (2 or more long-term health issues) were less likely to be referred than those without multimorbidity (OR: 0·55; [0·46, 0·68]; *p* < 0.001). Those with cognitive or physical disabilities were also less likely to be referred in the unadjusted model. The degree of hearing loss was significantly associated with likelihood for referral (*p* < 0·001), whereby adults with hearing thresholds worse than 90 dBHL were more likely to be referred than those with severe hearing loss, despite the latter adults still being eligible for referral (OR: 4·07; [2·16, 7·67]; *p* < 0.001). Finally, those adults seen at a centre specialising in implantation were more likely to be referred (OR: 5·49; [4·55, 6·62]; *p* < 0.001).

### Secondary outcome

#### Association of ethnicity and socioeconomic predictors on likelihood of discussion about referral after completing audiometric evaluation

Overall, only 35.7% (*n* = 2,204/6,171) of eligible adults had a documented discussion about their eligibility of being considered for surgical management of their hearing loss. The results of the logistic regression models for the association of socioeconomic and ethnicity factors are shown in **Table 3**. In the fully adjusted model, the deprivation index of adults was significantly associated with likelihood of being informed of their eligibility for referral (noted as “discussion”) (*p* < 0·001). Adults from the most deprived location were less likely to be discussed with than those from less deprived areas (deciles 4th–8th inclusive, where 10th is least deprived, **Table 3**). There were regional differences (*p* < 0·001), with London and the Northern regions having a lower OR for discussion compared to adults from the Midlands and East: North of England (OR: 0·74; [0·62, 0·89]; *p* = 0.001), London (OR: 0·44; [0·35, 0·56]; *p* < 0.001). In the fully adjusted model, ethnicity was significantly associated with likelihood of discussion (*p* < 0·001), whereby Asian (OR: 0·58; [0·43, 0·79]; *p* < 0.001) and black adults (OR: 0·56; [0·34, 0·92]; *p* = 0.021) were less likely to have a discussion compared to white counterparts.

**Table 3:** Results of simple and multivariable logistic regression analysis for the secondary outcome: whether patients had a discussion informing them that they were eligible for surgical management of their hearing loss, or not.

Characteristic	No. of events, *n/N* (%)	Simple logistic regression			Multivariable logistic adjusted model*		
		Beta	OR [95% CI]	*p-value*	Beta	OR [95% CI]	*p-value*
**Ethnicity and socioeconomic factors**							
**Race/ethnicity**				0·001			<0·001
White	1,671/4,679 (35.7)	Reference			Reference		
Asian	119/385 (30.9)	−0·22	0·81 [0·64, 1·01]	0·058	−0·54	0·58 [0·43, 0·79]	<0·001
Black	35/89 (39.3)	0·15	1·17 [0·76, 1·79]	0·482	−0·58	0·56 [0·34, 0·92]	0·021
Mixed	13/28 (46.4)	0·44	1·56 [0·74, 3·29]	0·242	0·02	1·02 [0·44, 2·39]	0·957
Other	126/280 (45.0)	0·39	1·47 [1·16, 1·88]	0·002	−0·11	0·90 [0·66, 1·23]	0·496
Missing	240/710 (33.8)	−0·08	0·92 [0·78, 1·09]	0·322	−0·46	0·63 [0·50, 0·80]	<0·001
IMD decile*				<0·001			<0·001
1st (most deprived)	172/608 (28.3)	Reference			Reference		
2nd	195/601 (32.4)	0·20	1·22 [0·95, 1·56]	0·116	0·27	1·31 [0·98, 1·75]	0·067
3rd	167/504 (33.1)	0·23	1·26 [0·97, 1·62]	0·081	0·24	1·27 [0·93, 1·72]	0·132
4th	229/553 (41.4)	0·58	1·79 [1·4, 2·29]	<0·001	0·69	1·99 [1·49, 2·66]	<0·001
5th	223/626 (35.6)	0·34	1·4 [1·1, 1·78]	0·006	0·56	1·75 [1·31, 2·33]	<0·001
6th	244/616 (39.6)	0·51	1·66 [1·31, 2·11]	<0·001	0·62	1·85 [1·39, 2·45]	<0·001
7th	232/618 (37.5)	0·42	1·52 [1·2, 1·94]	0·001	0·51	1·66 [1·25, 2·21]	<0·001
8th	252/652 (38.7)	0·47	1·6 [1·26, 2·02]	0·000	0·55	1·74 [1·31, 2·31]	<0·001
9th	233/645 (36.1)	0·36	1·43 [1·13, 1·82]	0·003	0·40	1·50 [1·12, 1·99]	0·006
10th (least deprived)	231/689 (33.5)	0·25	1·28 [1·01, 1·62]	0·042	0·39	1·47 [1·11, 1·96]	0·007
**Region**				<0·001			<0·001
Midlands and East	715/1,923 (37.2)	Reference			Reference		
London	248/654 (37.9)	0·03	1·03 [0·86, 1·24]	0·736	−0·82	0·44 [0·35, 0·56]	<0·001
North	331/1,189 (27.8)	−0·43	0·65 [0·56, 0·76]	<0·001	−0·30	0·74 [0·62, 0·89]	0·001
South	648/1,565 (414.4)	0·18	1·19 [1·04, 1·37]	0·011	0·02	1·02 [0·85, 1·22]	0·868
Scotland	52/228 (22.8)	−0·69	0·5 [0·36, 0·69]	<0·001	n/a		
Wales	183/400 (45.8)	0·35	1·42 [1·15, 1·77]	0·001	n/a		
Missing	27/212 (12.7)	−1·40	0·25 [0·16, 0·37]	<0·001	−2·33	0·10 [0·05, 0·19]	<0·001
**Healthcare factors**							
**Multimorbidity**				0·002			
No	1,024/2,784 (36.8)	Reference					
Yes	794/2,404 (33.0)	−0·17	0·85 [0·76, 0·95]	0·005			
Missing	386/983 (39.3)	0·11	1·11 [0·96, 1·29]	0·166			
**Cognitive disability**				<0·001			
No	1,787/4,924 (36.3)	Reference					
Yes	201/424 (47.4)	0·46	1·58 [1·30, 1·93]	<0·001			
Missing	216/822 (26.3)	−0·47	0·63 [0·53, 0·74]	<0·001			
**Physical disability**				0·005			
No	1,533/4,376 (35.0)	Reference					
Yes	311/759 (41.0)	0·25	1·29 [1·10, 1·51]	0·002			
Missing	360/1,036 (34.7)	−0·01	0·99 [0·86, 1·14]	0·864			
**Meningitis**				0·063			
No	1,733/4,816 (36.0)	Reference					
Yes	18/34 (52.9)	0·69	2·00 [1·02, 3·94]	0·044			
Missing	453/1,321 (34.3)	−0·07	0·93 [0·82, 1·06]	0·255			
**Visual impairment**				<0·001			
No	1,614/4,474 (36.1)	Reference					
Yes	342/861 (39.7)	0·16	1·17 [1·01, 1·36]	0·042			
Missing	248/836 (29.7)	−0·29	0·75 [0·64, 0·88]	<0·001			
**Learning disability**				0.002			
No	1,950/5,403 (36.1)	Reference					
Yes	67/150 (44.7)	0·36	1·43 [1·03, 1·98]	0·032			
Missing	187/618 (30.3)	−0·26	0·77 [0·64, 0·92]	0·004			
**Sex**				<0·001			
Male	1,067/3,355 (31.8)	Reference					
Female	1,127/2,787 (40.4)	0·38	1·46 [1·31, 1·62]	<0·001			
Missing	10/29 (34.5)	0·12	1·13 [0·52, 2·44]	0·758			
**Age**							
	Discussed 69 year; not discussed 75 year	−0·02	0·98 [0·98, 0·98]	<0·001			
**Hearing thresholds worse than 90 decibels hearing level**							
No	122/407 (30.0)	Reference					
Yes	2,082/5,764 (36.1)	0·28	1·32 [1·02, 1·64]	0·013			
**English as 1st language**				0·027			
No	156/382 (40.8)	Reference					
Yes	1,771/5,048 (35.1)	−0·24	0·78 [0·63, 0·97]	0·024			
Missing	277/741 (37.4)	−0·15	0·87 [0·67, 1·11]	0·260			
**Cochlear implant centre co-located**							
No	1,262/4,294 (29.4)	Reference					
Yes	942/1,877 (50.2)	0·88	2·42 [2·16, 2·71]	<0·001			
**Cochlear implant champion**							
No	426/1,431 (29.8)	Reference					
Yes	1,778/4,740 (37.5)	0·35	1·42 [1·25, 1·61]	<0·001			

* These models were run for patients with an English postcode only as IMD deciles are not comparable across the devolved nations; the majority (86%) of patients had an English postcode. Total included patients, *N* = 6,171.

CI, confidence interval; IMD, indices of multiple deprivation; OR, odds ratio.

### Association of other predictors on likelihood of discussion about referral after completing audiometric evaluation

In the unadjusted model, similar trends were identified as those seen with likelihood of referral after completing audiometric evaluation, whereby older adults were less likely to have a discussion of their eligibility (OR: 0·98; [0·98, 0·98]; *p* < 0.001). Female adults were more likely to be informed of their eligibility than male (OR: 1·46; [1·31, 1·62]; *p* < 0.001). Two site-specific factors were significant. Adults seen at a centre specialising in implants were more likely to be discussed (OR: 2.42; [2.16, 2.71]; *p* < 0.001). In addition, the presence of a cochlear implant champion at the hospital associated a highly significant benefit on having a discussion (OR: 1.42; [1.25, 1.61]; *p* < 0.001). All healthcare predictors were significantly associated with the outcome in the unadjusted regression model.

For exploratory and sensitivity analyses, see S3 Appendix and S1 and S2 Tables.

## Discussion

Only 9.7% (*n* = 546) of adults meeting pure tone audiometric threshold criteria for cochlear implantation assessment in a 6-month period in 2021 were referred for further evaluation, and 35.7% (*n* = 2,204) had a documented discussion about their eligibility for referral. Our hypothesis was upheld as there were disparities in the management of adults based on ethnic and socioeconomic factors, although the finding that males were less likely than females to be referred for assessment after audiometric evaluation for higher-level care was unexpected. Individuals from more deprived areas, specific regions, and older age groups were less likely to be referred. Moreover, adults from Asian and black backgrounds were less likely to have discussions informing them of their eligibility for cochlear implant assessment.

This study adds to the field of cochlear implant candidacy research by highlighting potential targets for increasing referral discussions for implants and suggests the need to explore the reasons behind the demographic, socioeconomic, and ethnic disparities to improve outcomes. According to the principles of shared decision-making and “no decision about me, without me” [10], all potentially eligible adults of cochlear implantation should be informed and given the option for assessment, and all members of the clinical team including audiologists and physicians should play a role in cochlear implant discussion and referral.

There was a trend of lower likelihood for discussion and referral after audiometric evaluation for implants among adults from socioeconomically deprived backgrounds. Similar findings have been observed in studies conducted worldwide. In Australia, recipients were more likely to live in the least 2 deprived deciles [7]. Similarly, in the US, adults with private insurance were 13 times more likely to receive cochlear implants compared to those under Medicare cover [11]. In an implant unit in the US, higher socioeconomic status patients were more likely to be implanted than those with lower socioeconomic status [8]. In countries with private healthcare systems, health-related financial inequalities may contribute to varied access across different sociodemographic groups. In nationalized healthcare systems like the UK, other factors such as patient and caregiver education, health literacy, social acceptance of cochlear implants, and unconscious bias may also play a role [12].

Geographic variations in management were observed, potentially influenced by factors such as clinician beliefs or service capacity. Adults from London were found to be the least likely to have discussions or referrals after audiometric evaluation for assessment compared to the East/Midlands. While population heterogeneity exists between regions, the fact that all identified adults were eligible for referral suggests differential management based on geographic location. Appreciating the underlying reasons can help inform loco-regional policies to establish coherent referral pathways. This in turn can enable monitoring of their effectiveness and highlight which aspects of the pathway may need additional targeted support and education.

Despite cochlear implant surgery being free at the point of access for adults in the UK, additional expenditures such as transportation, travel time or distance, parking for multiple appointments, as well as the potential need to take unpaid time away from employment, may deter some individuals. This study indicated that adults who had their referral candidacy confirmed with audiometric assessment at an implant centre were more likely to be referred, possibly due to geographical proximity between the patient’s home and the implant centre, but also possibly due to a greater intra-departmental appreciation of cochlear implantation as an option for these patients, and reduced administrative burden in referring these patients to colleagues in the same centre. In Australia, the majority of adult recipients lived near major urban centres [7]. Among American veterans, rural adults waited longer to obtain both hearing aids and implants, due to longer commutes and lower incomes [13]. This suggests the importance of centre location, and innovative approaches such as virtual consultations or remotely located audiologists to help redistribute implantation delivery and improve patient access [14].

The presence of a cochlear implant champion increased the likelihood of discussion of cochlear implant assessment referral eligibility based on pure tone audiometric criteria, but it did not translate into more referrals (in fact, marginally fewer patients were referred although *p* = 0.04). This suggests that not all eligible adults are suitable for various reasons, including audiological and healthcare factors, or patient choice. For a patient, the decision to undertake assessment and surgical management of their hearing loss is complex [5]. The champion program may need review to ensure clinicians have sufficient time to perform their role to aid identifying eligible adults and providing further information. Auditbase software has the capability to enable far more extensive coding of when referral discussions take place. This would allow clearer insight of where and when these discussions happen, although may require additional training locally, and additional time to undertake this extra task. Alternative diagnostic testing, such as speech testing, could aid the clinical decision-making process [15] and explain regional variations in management.

Adults from Asian and black backgrounds were less likely to have a discussion informing them of their eligibility for referral after audiometric evaluation. Variations in clinician referral rates by ethnicity have also been shown to affect referrals for a range of hearing and non-hearing services [16,17]. A study in the US also found that African-American and Asian patients were less likely to accept proceeding with cochlear implantation [18]. There may be factors such as language barriers or clinician biases which have not been explored by our work. It is noted that while our data suggested that not having English as a first language did not significantly impact the discussion of cochlear implants, it did correlate with reduced referral rates. This is consistent with a study from the USA which revealed that non-English speaking patients received fewer referrals compared to English-speaking patients [19]. This may suggest difficulties in adequately explaining cochlear implantation as an option or cultural differences in approaching hearing loss treatment. Compared to white counterparts, minority ethnic groups are less likely to use hearing aids [16] and report lower familiarity with cochlear implants [20].

It is important to note that this study focused on individuals who had already sought hearing healthcare, potentially underrepresenting Asian and black communities. Reasons for underrepresentation may include cultural norms of elder care that mitigate the impact of hearing loss, systemic healthcare access barriers in minority ethnic groups, or the overrepresentation of younger adults who migrated to the UK. Encouraging older Asian or black communities to seek hearing tests is crucial moving forward and an area for future research.

Older adults were less likely to be referred or informed of their eligibility for referral for assessment. Older patients declining cochlear implantation has also been found by a study in the US [18]. Age discrimination may play a role in perceiving hearing loss as an inevitable consequence of ageing [21]. However, evidence suggests improved speech scores and quality of life following implantation in adults aged 65 to 80 years, with few complications when managed appropriately [22]. Age was positively correlated with multimorbidity (i.e., older patients were more likely to suffer from multimorbidity) and may play a role in patients’ likelihood to accept a referral for cochlear implant assessment. A recent population cohort study examining multimorbidity in surgery indicated that 11.2% of patients undergoing elective surgery had multimorbidity yet multimorbid patients accounted for 50.2% of deaths after elective spells [23]. Recognising they are at higher risk of poor outcomes may influence the decision-making process in declining assessment even at this early stage in the patient pathway.

Men were also found to be less likely to be referred or informed of their eligibility for referral for assessment. Interestingly, a study in the US showed that men were not less likely to proceed with surgery compared to women [18]. The influence and magnitude of our effect size for sex was an unanticipated finding. This may be explained by the divergent health-seeking behaviours that exist between men and women. Previous studies on gender differences in the patterns of use of conventional hearing aids have found that hearing-impaired men were less likely to be acceptant of hearing loss, had lower motivation to seek hearing rehabilitation, and were more concerned about their appearance [24,25]. Despite this, few sex-specific initiatives for the management of hearing loss exist.

The degree of hearing loss was associated with likelihood for referral after audiological evaluation; adults with hearing thresholds worse than 90 dBHL (i.e., profound loss) were more likely to be referred than those with severe hearing loss, despite the latter adults still meeting pure tone threshold criteria for referral. This suggests that eligible adults with severe hearing loss, and/or their clinicians, may perceive that they are managing their hearing loss or that they lack awareness of the latest referral guidelines [3]. There may be a concern that implants may sacrifice their residual hearing; however, appropriate counselling about hearing preservation surgery can be done by a cochlear implant team [26].

The strengths of this study include the large sample size, focus on adults (often work focuses on the paediatric population), and the inclusion of all eligible candidates meeting pure tone audiological threshold criteria, rather than those who have already been referred for assessment. There are a number of limitations to be discussed.

Further weaknesses include the observational retrospective nature, dependence on accurate documentation, and potential underrepresentation of certain regions [9] or demographic groups. The models used assume correlations and do not predict causality. There was a reliance upon accurate documentation of patient discussions and it is conceivable that discussions were had for some adults yet not recorded. Furthermore, without the ability to contact patients directly, gender identity was determined by accuracy of NHS sex categorisation. The study was conducted during the COVID-19 pandemic, which may have affected referral rates (S4 Appendix). A further weakness of this study is that it only captures patient assessments performed in secondary care and was not able to capture those performed in other settings, such as high street audiology assessments.

This study only includes individuals who have successfully accessed secondary healthcare and may exclude those encountering barriers such as socioeconomic, systemic, or cultural challenges in accessing primary healthcare. This approach risks disproportionately representing the perspectives of those with easier access, reinforcing disparities and impeding a comprehensive understanding of diverse healthcare needs. To mitigate this, future research should consider approaches to actively incorporate individuals underrepresented due to barriers in seeking healthcare. We also relied on the electronic health records of the services referring to the cochlear implant team and cannot confirm that patients attended and completed a cochlear implant assessment which could give further information about patient barriers to referral rather than referrer barriers.

Speech audiometry, which forms part of NICE cochlear implant candidacy criteria, is not routinely performed in the UK as part of a standard hearing assessment and was therefore not included in our data collection. Clinicians should consider requesting this to aid their decision-making process regarding discussion and possible referral for cochlear implantation.

The effects of untreated or undertreated hearing loss can be diverse, being associated with depression, social isolation, risk of hospitalisation, and mortality [27–30]. There is a strong link between hearing loss and cognitive decline, including dementia. Although the exact cause of this link is undergoing research, treatment of auditory impairment could delay or prevent neurodegenerative changes if they occur due to altered sensory input [31]. An analysis of the UK Biobank data has shown dementia is more likely to develop in black participants than white which reinforces consideration of targeting potential modifiable risk factors of cognitive decline in black ethnic groups [32]. Thus, the results of this research imply that differences at this early stage in candidacy discussions may have substantial implications for further health issues, above and beyond that of hearing loss.

Future research should adopt a nuanced approach to investigate the reasons behind the ethnic, socioeconomic, and other demographic differences in assessment. The referral pathway is likely to be influenced by various factors related to clinicians, patients, and systems. However, the specific contributions of each stakeholder remain unclear in the existing research. Conducting qualitative assessments involving key stakeholders, such as patients and clinicians, could shed light on these contributions. This approach may not only help investigate the dynamics involved but also identify adaptable strategies to enhance accessibility and pinpoint areas for targeted educational initiatives. Utilising information technology systems in audiology can help identify eligible adults. Efforts are underway to create an automatic pop-up identification tool in the next published version of Auditbase. This should be used alongside running regular audit reports and contacting patients accordingly should be encouraged in all UK hospitals.

In conclusion, our findings indicate that a significant number of eligible adults with severe-to-profound hearing loss in Great Britain are not being appropriately referred after meeting pure tone audiometric threshold criteria for cochlear implant assessment or informed about their eligibility for referral. This study highlights that there are notable disparities in patient management based on socioeconomic status, ethnicity, and sex. With this knowledge, clinicians and policymakers may wish to explicitly target these underrepresented patient groups via support and education to those teams directly caring for them to improve discussion and referral rates for cochlear implants. While it is acknowledged that not all eligible adults may opt for surgery, it is crucial that all patients are made aware of their eligibility for further assessment for this potential life-altering intervention. Further research is necessary to understand and address these disparities, with a focus on developing tools to ensure informed decision-making, educating hearing healthcare providers, and investigating the reasons behind cochlear implant declination and the underrepresentation of older ethnic minority groups in seeking hearing assessments in secondary care. The aim is to enhance equitable access to hearing loss treatment.

## Supporting information

S1 Checklist STROBE checklist. (DOCX)

S1 Appendix De-duplication methods. (DOCX)

S2 Appendix Further information pertaining to indices of multiple deprivation (IMD). (DOCX)

S3 Appendix Exploratory and sensitivity analyses. (DOCX)

S4 Appendix COVID restrictions. (DOCX)

S1 Flowchart Flow chart of included patients. (DOCX)

S1 Table Impact of IMD subdomains upon likelihood of referral (patients with postcodes in England only) (multivariable logistic regression). Odds ratio (OR), confidence interval (CI), indices of multiple deprivation (IMD), income deprivation affecting older people index (IDAOPI). (DOCX)

S2 Table Impact of removing patients with missing data, even in just one variable, upon the primary outcome (likelihood of referral) (included *n* = 3,778; missing *n* = 2,212; CI patients *n* = 246) (multivariable adjusted logistic regression model). Odds ratio (OR), confidence interval (CI), indices of multiple deprivation (IMD). (DOCX)

S1 Membership of INTEGRATE Collaborative authors. (XLSX)

## Abbreviations

BCIG: British Cochlear Implant Group
BSO: British Society of Otology
CI: confidence interval
IDAOPI: income deprivation affecting older people index
IMD: Indices of Multiple Deprivation
NHS: National Health Service
NICE: National Institute for Health and Care Excellence
OR: odds ratio
SD: standard deviation

## References

[1] Office of National Statistics. Population estimates for the UK, England and Wales, Scotland and Northern Ireland. Available from: https://www.ons.gov.uk/peoplepopulationandcommunity/populationandmigration/populationestimates/bulletins/annualmidyearpopulationestimates/mid2021. Accessed 2023 May 31.

[2] Department for Work & Pensions. What works: Hearing loss and employment. Available from: https://www.england.nhs.uk/wp–content/uploads/2017/09/hearing–loss–what–works–guide–employment.pdf. Accessed 2023 May 31.

[3] National Institute for Health and Care Excellence (NICE). Cochlear implants for children and adults with severe to profound deafness (TA566). Available from: https://www.nice.org.uk/guidance/ta566/resources/cochlear–implants–for–children–and–adults–with–severe–to–profound–deafness–pdf–82607085698245. Accessed 2023 May 31. 40902019

[4] Andries E, Gilles A, Topsakal V, Vanderveken OM, Van de Heyning P, Van Rompaey V, et al. Systematic Review of Quality of Life Assessments after Cochlear Implantation in Older Adults. Audiol Neurotol. 2021;26(2):61–75. doi: 10.1159/000508433 32653882

[5] Bierbaum M, McMahon CM, Hughes S, Boisvert I, Lau AYS, Braithwaite J, et al. Barriers and Facilitators to Cochlear Implant Uptake in Australia and the United Kingdom. Ear Hear. 2020;41(2):374–385. doi: 10.1097/AUD.0000000000000762 31356385

[6] Raine C. Cochlear implants in the United Kingdom: Awareness and utilization. Cochlear Implants Int. 2013;14:S32–S37. doi: 10.1179/1467010013Z.00000000077 23453150 PMC3663289

[7] Cheung LL, Fowler A, Hassarati R.T, Birman CS. Distance and Socieoeconomic Status as Barriers to Cochlear Implantation. Otol Neurotol. 2023;44:134–140. doi: 10.1097/MAO.0000000000003765 36624590

[8] Quimby AE, Venkatesh S, Corsten M, McDonald JT, Hwa TP, Bigelow DC. Socioeconomic Status Among Cochlear Implant Candidates and Association With Surgical Pursuance. JAMA Otolaryngol Head Neck Surg. 2023;10:891–8. doi: 10.1001/jamaoto.2023.2217 37615991 PMC10450586

[9] Abel GA, Barclay ME, Payne RA. Adjusted indices of multiple deprivation to enable comparisons within and between constituent countries of the UK including an illustration using mortality rates. BMJ Open. 2016;6:e012750. doi: 10.1136/bmjopen-2016-012750 27852716 PMC5128942

[10] Department of Health. Equity and excellence: liberating the NHS. Norwich: Stationery Office; 2010.

[11] Tolisano AM, Schauwecker N, Baumgart B, Whitson J, Kutz JW Jr, Isaacson B, et al. Identifying Disadvantaged Groups for Cochlear Implantation: Demographics from a Large Cochlear Implant Program. Ann Otol Rhinol Laryngol. 2020;129:347–354. doi: 10.1177/0003489419888232 31735055

[12] Schuh M, Bush ML. Defining Disparities in Cochlear Implantation through the Social Determinants of Health. Semin Hear. 2021;42:321–330. doi: 10.1055/s-0041-1739282 34912160 PMC8660167

[13] Hixon B, Chan S, Adkins M, Shinn JB, Bush ML. Timing and Impact of Hearing Healthcare in Adult Cochlear Implant Recipients: A Rural–Urban Comparison. Otol Neurotol. 2016;37:1320–1324. doi: 10.1097/MAO.0000000000001197 27636389 PMC5027983

[14] Nassiri AM, Saoji AA, DeJong MD, Tombers NM, Driscoll CLW, Neff BA, et al. Implementation Strategy for Highly–Coordinated Cochlear Implant Care With Remote Programming: The Complete Cochlear Implant Care Model. Otol Neurotol. 2022;43:e916–923. doi: 10.1097/MAO.0000000000003644 35970171 PMC9394487

[15] Parmar BJ, Rajasingam SL, Bizley JK, Vickers DA. Factors Affecting the Use of Speech Testing in Adult Audiology. Am J Audiol. 2022;31:528–540. doi: 10.1044/2022_AJA-21-00233 35737980 PMC7613483

[16] Nieman CL, Marrone N, Szanton SL, Thorpe RJ, Lin FR. Racial/Ethnic and Socioeconomic Disparities in Hearing Health Care Among Older Americans. J Aging Health. 2016;28:68–94. doi: 10.1177/0898264315585505 25953816 PMC4826391

[17] Goulart BHL, Reyes CM, Fedorenko CR, Mummy DG, Satram-Hoang S, Koepl LM, et al. Referral and Treatment Patterns Among Adults With Stages III and IV Non–Small–Cell Lung Cancer. J Oncol Pract. 2013;9:42–50. 23633970 10.1200/JOP.2012.000640 PMC3545662

[18] Kaul VF, Dzubara BP, Munjal V, Katta J, Adunka OF, Ren Y. Investigating Deferral Rates in Cochlear Implantation: How Often Do Candidates Defer and Why? Otol Neurotol. 2023;45(1):10–97. doi: 10.1097/MAO.0000000000004045 38013485

[19] Greiner RC, Rubinstein JT, Kohlberg GD. Investigating Socioeconomic Barriers to Cochlear Implantation. Otol Neurotol. 2023;44:e660–e666. doi: 10.1097/MAO.0000000000003985 37604510

[20] Marinelli JP, Sydlowski SA, Carlson ML. Cochlear Implant Awareness in the United States: A National Survey of 15,138 Adults. Semin Hear. 2022;43:317–323. doi: 10.1055/s-0042-1758376 36466559 PMC9715307

[21] Marinelli JP, Carlson ML. Barriers to Access and Health Care Disparities Associated With Cochlear Implantation Among Adults in the United States. Mayo Clin Proc. 2021;96:547–549. doi: 10.1016/j.mayocp.2020.08.027 33673908

[22] Orabi AA, Mawman D, Al–Zoubi F, Saeed SR, Ramsden RT. Cochlear implant outcomes and quality of life in the elderly: Manchester experience over 13 years. Clin Otolaryngol. 2006;31:116–122. doi: 10.1111/j.1749-4486.2006.01156.x 16620330

[23] Fowler AJ, Hussein Wahedally MA, Abbott TEF, Prowle JR, Cromwell DA, Pearse RM. Long-term disease interactions amongst surgical patients: a population cohort study. Br J Anaesth. 2023;131:407–417. doi: 10.1016/j.bja.2023.04.041 37400340 PMC10375505

[24] Staehelin K, Bertoli S, Probst R, Schindler C, Dratva J, Stutz EZ. Gender and Hearing Aids: Patterns of Use and Determinants of Nonregular Use. Ear Hear. 2011;32:e26–e37. doi: 10.1097/AUD.0b013e3182291f94 21795978

[25] Saunders GH, Cienkowski KM, Forsline A, Fausti S. Normative Data for the Attitudes towards Loss of Hearing Questionnaire. J Am Acad Audiol. 2005;16:637–652. doi: 10.3766/jaaa.16.9.2 16515136

[26] Harrison L, Manjaly JG, Ellis W, Lavy JA, Shaida A, Khalil SS, et al. Hearing Preservation Outcomes With Standard Length Electrodes in Adult Cochlear Implantation and the Uptake of Electroacoustic Stimulation. Otol Neurotol. 2020;41:1060–1065. doi: 10.1097/MAO.0000000000002702 32569131

[27] Yeo BSY, Song HJJMD, Toh EMS, Ng LS, Ho CSH, Ho R, et al. Association of Hearing Aids and Cochlear Implants With Cognitive Decline and Dementia: A Systematic Review and Meta–analysis. JAMA Neurol. 2022;80(2):134–141. 10.1001/jamaneurol.2022.4427 PMC9856596 36469314

[28] Shukla A, Harper M, Pedersen E, Goman A, Suen JJ, Price C, et al. Hearing Loss, Loneliness, and Social Isolation: A Systematic Review. Otolaryngol Head Neck Surg. 2020;162(5):622–633. doi: 10.1177/0194599820910377 32151193 PMC8292986

[29] Genther DJ, Frick KD, Chen D, Betz J, Lin FR. Association of Hearing Loss With Hospitalization and Burden of Disease in Older Adults. JAMA. 2013;309:2322–2324. doi: 10.1001/jama.2013.5912 23757078 PMC3875309

[30] Tan BKJ, Ng FYC, Song HJJMD, Tan NKW, Ng LS, Loh WS. Associations of Hearing Loss and Dual Sensory Loss With Mortality: A Systematic Review, Meta–analysis, and Meta–regression of 26 Observational Studies With 1,213,756 Participants. JAMA Otolaryngol Head Neck Surg. 2022;148:220–234. 34967895 10.1001/jamaoto.2021.3767 PMC8719275

[31] Tarawneh HY, Jayakody DMP, Sohrabi HR, Martins RN, Mulders WHAM. Understanding the Relationship Between Age–Related Hearing Loss and Alzheimer’s Disease: A Narrative Review. J Alzheimers Dis Rep. 2022;6:539–56. doi: 10.3233/ADR-220035 36275417 PMC9535607

[32] Mukadam N, Marston L, Lewis G, Livingston G. Risk factors, ethnicity and dementia: A UK Biobank prospective cohort study of White, South Asian and Black participants. PLoS ONE. 2022;17:e0275309. doi: 10.1371/journal.pone.0275309 36223334 PMC9555673

