# Supplementary material for: Socioeconomic and ethnic disparities associated with access to cochlear implantation for severe-to-profound hearing loss: A multicentre observational study of UK adults
Source: PLoS Med. 2024 Apr 4;21(4):e1004296. doi: 10.1371/journal.pmed.1004296 (PMC10994380; doi:10.1371/journal.pmed.1004296)
Supplement: S1 Appendix — (DOCX) [file pmed.1004296.s002.docx]

**S1 Appendix. De-duplication methods**

To facilitate identification of duplicate adults (i.e., those who may have had audiometric testing at more than one site during the study period), local teams used a one–way anonymisation process to encrypt adults’ National Health Service (NHS) numbers, which was then trimmed to a unique 20–character hexadecimal code. This made decryption of the unique code impossible but ensured the same NHS number always produced the same unique code. In this way, the project management team was able to anonymously identify duplicate patient records across participating sites.
