# Supplementary material for: Socioeconomic and ethnic disparities associated with access to cochlear implantation for severe-to-profound hearing loss: A multicentre observational study of UK adults
Source: PLoS Med. 2024 Apr 4;21(4):e1004296. doi: 10.1371/journal.pmed.1004296 (PMC10994380; doi:10.1371/journal.pmed.1004296)
Supplement: S2 Appendix — (DOCX) [file pmed.1004296.s003.docx]

**S2 Appendix. Further information pertaining to indices of multiple deprivation (IMD)**

In the UK, separate IMD documents are available across the four devolved nations, and direct comparisons between the four constituent nations of the UK are not possible[8]. In England, the IMD (2019) is an open–access, official measure of relative deprivation for small neighbourhoods, and ranks every small area from one (most deprived area) to 32,844 (least deprived area) [1]. Deprivation ‘deciles’ are published by dividing the 32,844 small neighbourhoods into ten equal groups, where one is the most deprived decile, and ten is the least deprived decile. The rank is calculated based on performance in seven deprivation domains: income, employment, education, health and disability, crime, barriers to housing and services, and living environment. Further supplementary information is provided regarding the income deprivation among older people (IDAOPI). The seven deprivation score breakdowns are not available for Scotland, Wales, or Northern Ireland.

**Reference**

1. Ministries of Housing, Communities, and Local Government. English Indices of Deprivation 2019. 2019. Available from: https://imd-by-postcode.opendatacommunities.org/imd/2019
