# Supplementary material for: Socioeconomic and ethnic disparities associated with access to cochlear implantation for severe-to-profound hearing loss: A multicentre observational study of UK adults
Source: PLoS Med. 2024 Apr 4;21(4):e1004296. doi: 10.1371/journal.pmed.1004296 (PMC10994380; doi:10.1371/journal.pmed.1004296)
Supplement: S3 Appendix — (DOCX) [file pmed.1004296.s004.docx]

**S3 Appendix. Exploratory and sensitivity analyses**

*Exploratory analyses*

Multivariable logistic regression was repeated for the primary outcome, using the fully adjusted analysis, to explore aspects of the hearing healthcare relevant IMD sub–domains for adults with an English postcode. Only the Income Deprivation Affecting Older People Index (IDAOPI) was associated with the likelihood of referral, whereby adults from less deprived areas were more likely to be referred. This association was strongest in the 5^th^–6^th^ and 8^th^–9^th^ deciles (**S1 Table**). Regional health, education, barriers to housing and services were not seen to affect the likelihood of referral.

*Sensitivity analyses*

Multivariable logistic regression was repeated for the primary outcome, excluding adults with any missing data (even in just one category). This led to 3778, rather than 6236, adults being included in the model. Predictors for non–referral were similar: adults from more deprived locations, adults from London or the North, those not seen at a Cochlear Implant centre, older adults, those with multiple medical conditions, those with hearing thresholds better than 90dBHL, and men (**S2 Table**).
