# Supplementary material for: Socioeconomic and ethnic disparities associated with access to cochlear implantation for severe-to-profound hearing loss: A multicentre observational study of UK adults
Source: PLoS Med. 2024 Apr 4;21(4):e1004296. doi: 10.1371/journal.pmed.1004296 (PMC10994380; doi:10.1371/journal.pmed.1004296)
Supplement: S4 Appendix — (DOCX) [file pmed.1004296.s005.docx]

**S4 Appendix. COVID restrictions**

During the time period from which data was collected, COVID-19 restrictions were lifted by the UK government. On 19^th^ July 2021, all restrictions were lifted. While face masks were recommended, they were not required by law, although in medical settings their use was continued. Restrictions were re-implemented on 15^th^ December 2021, with the government’s “Plan B rules” that encouraged people to work from home and face masks became compulsory again in most indoor settings. Secondary care hospital appointments in all centres from which data were collected were open and functioning during this time period, but new concerns of inbound Winter pressures and restrictions may have influenced referral rates. Additionally, continued compulsory use of face masks in hospitals may have had an impact on communication with patients, although many audiology departments made use of clear face coverings for those with difficulty hearing.
