# Supplementary figures and images for: Socioeconomic and ethnic disparities associated with access to cochlear implantation for severe-to-profound hearing loss: A multicentre observational study of UK adults
Source: PLoS Med. 2024 Apr 4;21(4):e1004296. doi: 10.1371/journal.pmed.1004296 (PMC10994380; doi:10.1371/journal.pmed.1004296)

**S1 Flowchart.** Flowchart of included patients

**
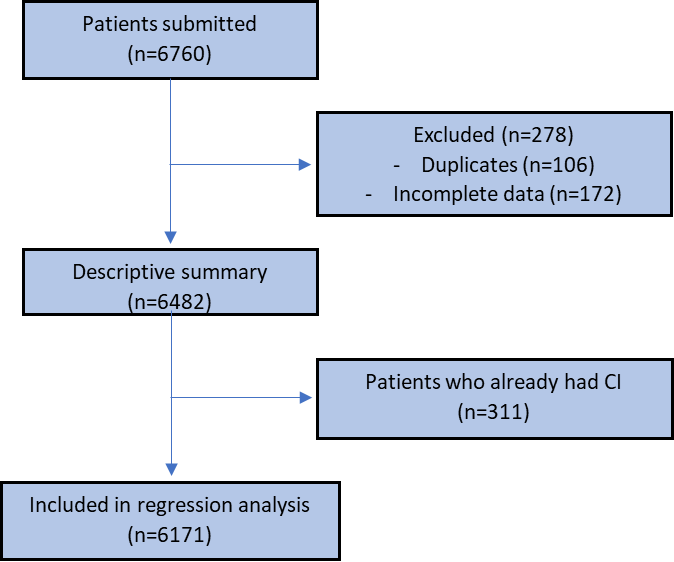
**

Supplement: S1 Flowchart — (DOCX) [file pmed.1004296.s006.docx]
