# Supplementary material for: Socioeconomic and ethnic disparities associated with access to cochlear implantation for severe-to-profound hearing loss: A multicentre observational study of UK adults
Source: PLoS Med. 2024 Apr 4;21(4):e1004296. doi: 10.1371/journal.pmed.1004296 (PMC10994380; doi:10.1371/journal.pmed.1004296)
Supplement: S1 Table — (DOCX) [file pmed.1004296.s007.docx]

**S1 Table.** Impact of IMD sub–domains upon likelihood of referral (patients with postcodes in England only) (multivariable logistic regression). Odds ratio (OR), Confidence interval (CI), Indices of multiple deprivation (IMD), Income deprivation affecting older people index (IDAOPI)

| **Characteristic** | **Likelihood of Referral** | | |
| --- | --- | --- | --- |
|  | **Beta** | **OR [95% CI]** | ***p–value*** |
| **IMD IDAOPI decile*** | |  | <0·001 |
| 1^st^ (most deprived) | Reference |  |  |
| 2^nd^ | –0·23 | 0·79 [0·48,1·32] | 0·370 |
| 3^rd^ | –0·12 | 0·89 [0·54,1·46] | 0·647 |
| 4^th^ | 0·34 | 1·41 [0·88,2·25] | 0·153 |
| 5^th^ | 0·59 | 1·80 [1·17,2·78] | 0·008 |
| 6^th^ | 0·48 | 1·62 [1·05,2·52] | 0·031 |
| 7^th^ | 0·15 | 1·16 [0·74,1·82] | 0·518 |
| 8^th^ | 0·41 | 1·51 [0·98,2·31] | 0·061 |
| 9^th^ | 0·48 | 1·61 [1·05,2·46] | 0·028 |
| 10^th^ (least deprived) | 0·07 | 1·06 [0·68,1·67] | 0·776 |
|  |  |  |  |
