# Supplementary material for: Socioeconomic and ethnic disparities associated with access to cochlear implantation for severe-to-profound hearing loss: A multicentre observational study of UK adults
Source: PLoS Med. 2024 Apr 4;21(4):e1004296. doi: 10.1371/journal.pmed.1004296 (PMC10994380; doi:10.1371/journal.pmed.1004296)
Supplement: S2 Table — (DOCX) [file pmed.1004296.s008.docx]

**S2 Table.** Impact of missing data.

**S2a.** Impact of removing patients with missing data, even in just one variable, upon the primary outcome (likelihood of referral) (included n=3959; missing n=2212; CI patients n=311) (multivariable adjusted logistic regression model). Odds ratio (OR), Confidence interval (CI), Indices of multiple deprivation (IMD)

| **Characteristic** | **Likelihood of Referral** | | |
| --- | --- | --- | --- |
|  | **Beta** | **OR [95% CI]** | ***p–value*** |
| **IMD decile*** |  |  | <0·001 |
| 1^st^ (most deprived) | Reference |  |  |
| 2^nd^ | 0·61 | 1·84 [0·99,3·42] | 0·054 |
| 3^rd^ | 0·51 | 1·66 [0·86,3·19] | 0·129 |
| 4^th^ | 0·78 | 2·18 [1·17,4·06] | 0·014 |
| 5^th^ | 0·62 | 1·85 [0·99,3·47] | 0·055 |
| 6^th^ | 1·18 | 3·25 [1·79,5·91] | <0·001 |
| 7^th^ | 0·54 | 1·72 [0·92,3·23] | 0·091 |
| 8^th^ | 0·63 | 1·87 [1·00,3·49] | 0·049 |
| 9^th^ | 0·16 | 1·17 [0·60,2·28] | 0·642 |
| 10^th^ (least deprived) | 0·48 | 1·61 [0·86,3·03] | 0·135 |
|  |  |  |  |
| **Region** |  |  | <0·001 |
| Midlands & East | Reference |  |  |
| London | –0·65 | 0·53 [0·35,0·78] | 0·001 |
| North | –0·44 | 0·64 [0·43,0·96] | 0·030 |
| South | 0·07 | 1·07 [0·79,1·45] | 0·652 |
|  |  |  |  |
| **Race/ethnicity** |  |  | 0·001 |
| White | Reference |  |  |
| Asian |  |  |  |
| Black |  |  |  |
| Mixed |  |  |  |
| Other |  |  |  |
|  |  |  |  |

**S2b.** Missing data (missing at random) replaced using multiple imputation (automated multiple imputation method in SPSS).

| **Characteristic** | **Likelihood of Referral** | | |
| --- | --- | --- | --- |
|  | **Beta** | **OR [95% CI]** | ***p–value*** |
| **IMD decile*** |  |  | <0·001 |
| 1^st^ (most deprived) | Reference |  |  |
| 2^nd^ | 0.42 | 1.52 [0.91, 2.54] | 0·112 |
| 3^rd^ | 0.51 | 1.66 [0.98, 2.81] | 0·061 |
| 4^th^ | 0.79 | 2.21 [1.34, 3.65] | 0·002 |
| 5^th^ | 0.69 | 1.99 [1.21, 3.29] | 0·007 |
| 6^th^ | 1.01 | 2.73 [1.68, 4.44] | <0·001 |
| 7^th^ | 0.44 | 1.55 [0.93, 2.60] | 0·094 |
| 8^th^ | 0.78 | 2.18 [1.33, 3.57] | 0·002 |
| 9^th^ | 0.17 | 1.19 [0.69, 2.03] | 0·533 |
| 10^th^ (least deprived) | 0.34 | 1.41 [0.84, 2.36] | 0·196 |
|  |  |  |  |
| **Region** |  |  |  |
| Midlands & East | Reference |  |  |
| London | -1.09 | 0.34 [0.24, 0.46] | <0.001 |
| North | -0.68 | 0.50 [0.36, 0.70] | <0.001 |
| South | -0.24 | 0.79 [0.62, 1.00] | 0·052 |
|  |  |  |  |
| **Race/ethnicity** |  |  |  |
| White | Reference |  |  |
| Asian |  |  |  |
| Black |  |  |  |
| Mixed |  |  |  |
| Other |  |  |  |
|  |  |  |  |
